# Molecular Mechanism of *Cuscuta* Haustorium Specialization Inferences from Transcriptome and Metabolome Analysis

**DOI:** 10.3390/metabo15030172

**Published:** 2025-03-03

**Authors:** Xingpan Meng, Ning Lv, Xinglin Wang, Qihang Zhou, Xu Zhang, Ximin Zhang, Zhengdong Zhang, Lunxian Liu, Tie Shen

**Affiliations:** 1 Key Laboratory of National Forestry and Grassland Administration on Biodiversity Conservation in Karst Mountainous Areas of Southwestern China, Engineering Research Center of Carbon Neutrality in Karst Areas, Ministry of Education, Key Laboratory of Environment Friendly Management on High Altitude Rhododendron Diseases and Pests, Institutions of Higher Learning in Guizhou Province, School of Life Science, Guizhou Normal University, Guiyang 550025, China; 222100100438@gznu.edu.cn (X.M.); lvning@gznu.edu.cn (N.L.); 232100100407@gznu.edu.cn (X.W.); zhxm409@gznu.edu.cn (X.Z.); 2 School of Cyber Sciences, Guizhou Normal University, Guiyang 550025, China; sail@gznu.edu.cn; 3 Guizhou Caohai Wetland Ecosystem National Observation and Research Station, Guizhou Academy of Forestry Sciences, Guiyang 550001, China; 232200101557@gznu.edu.cn; 4 College of Computer Science, Guiyang University, Guiyang 550001, China; zzd@gznu.edu.cn

**Keywords:** *Cuscuta chinensis*, aspirators, distal stem, metabolomics, transcriptomics

## Abstract

Background: *Cuscuta australis R. Br.* is a parasitic herbaceous plant that obtains nutrients by forming specialized structures called haustoria to invade host plants. Methods: In this study, we elucidated the differences in the gene expression regulation and metabolic characteristics between *Cuscuta australis* and *Glycine max* (*Glycine max* (L.) *Merr. Var Williams*) through comprehensive transcriptomic and metabolomic analyses. Results: The results demonstrated significant differences in the gene expression and metabolic features between the haustorium and the distal stem segments. The differentially expressed genes absorbed by *Cuscuta australis* from the soybean host influence amino acid metabolism, and the expression of the S-adenosylmethionine decarboxylase gene may affect the production of 5′-methylthioadenosine. A high expression of the chalcone synthase enzyme could lead to an increased daidzein content. Many *Glycine max* genes were also integrated into *Cuscuta australis* within the haustorium. Conclusions: This study systematically analyzed, for the first time, the significant differences in gene expression and metabolic characteristics between the haustoria and distal stem segments of *Cuscuta*. It also explored the nutrient absorption mechanisms of the host plant. Additionally, the research discovered that *Cuscuta* can absorb a substantial amount of host genes and adapt to its parasitic lifestyle through differential gene expression and metabolic changes. These findings provide important insights into the parasitic mechanisms of *Cuscuta australis* and lay the foundation for the development of effective control strategies.

## 1. Introduction

*Cuscuta australis* R. Br. has a wide range of hosts and strong parasitic ability, and its stem segment will form a sucker to invade the host plant and finally connect with the vascular tissue of the host plant [1]. As a parasitic plant, *Cuscuta australis R. Br.* relies on its haustoria and stems as the core of its parasitic mechanism. The haustorium is responsible for substance exchange with the host plant, and studying it can reveal its parasitic strategies and mechanisms for nutrient acquisition. The stem, related to growth and spread, is crucial for studying parasitic behavior. Both provide important avenues for exploring the dynamics of parasitic biology and host–parasite interactions.

In recent years, researchers have analyzed the structure of the “aspirator”, including the molecular anatomy of the aspirator development [2], the morphogenesis of the aspirator [3], the influencing factors of the aspirator formation [4,5,6], the effects of hormones on the aspirator [7,8], etc. As a model organism for the development of stem holoparasitic plant suckers [9], it is of great significance to study the occurrence and function of *Cuscuta*. The snicker can absorb various nutrients needed for its growth and development, including plant hormones, proteins, and RNA [2,8,10], to ensure survival. The exchange of such substances between two species is called cross-species transmission [11,12].

A large number of studies on the adaptation, interaction, and evolution of parasitic systems in cross-species transmission have shown that cross-species transmission is essential for the growth and development of parasitic plants [13,14,15]. At the same time, with the rapid improvement of omics technology, multi-omics combined analysis can provide better and more comprehensive results, which is an effective method to reveal the complex metabolic biosynthesis and response genes between samples. Kumar and Amir (2021) used metabolomics to analyze the metabolites of the three organs of the sucker, stem, and flower of the original dodder when it was parasitic in different hosts, and the results showed that the metabolites of the three organs of the original dodder were different in different hosts [16]; Ichihashi et al. (2015) used transcriptomics to elaborate on the “suckers” of parasitic plants, revealing the uniqueness of parasitic plants relative to autotrophic plants, and whether or not parasitic *Cuscuta* has a significant impact on the defense genes and pathways of white clover [17,18]. At the same time, the joint analysis of parasitic plants via metabolomics and transcriptomics has also been reported, such as the root and sucker of *Phylum* spp. [19] revealing a unique metabolic profile, containing a variety of fatty acids in the roots, and the combined analysis of *Cuscuta japonica* in both host and non-host plants, and found that there were significant differences in the synthesis of phenylpropanes. Research by Lehtonen indicates that Late Goldenrod (*Solidago gigantea*) can acquire fungal toxins from host plants, such as perennial ryegrass or fescue, that are infected by symbiotic endophytic fungi like Epichloë [20].

Currently, the genomes of *C. australis* and *G. max* (*Glycine max* (L.) *Merr. Var Williams*) have been sequenced and published. By integrating the genomic data of these two species, it is possible to analyze the transcriptome information that *C. australis* acquires from its host soybean. Therefore, we performed a combined transcriptomic and metabolomic analysis of the haustoria (XQ) and stems (YD) of the soybean (Qiandou)-Cuscuta (*C. australis*) system. Although previous studies have made preliminary explorations into the parasitic mechanisms of Cuscuta, most of them have focused solely on its morphological characteristics or single-omics level analysis. Building on past research, this study innovatively employed a combined transcriptomic and metabolomic analysis to systematically compare the differences in the gene expression and metabolic characteristics between the haustoria and distal stem segments of Cuscuta. Compared to previous studies, this research not only reveals the mechanisms of gene absorption and utilization from the host during parasitism but also further elucidates the complexity of its metabolic regulation. By dissecting the differential gene distribution and its impact on metabolomic characteristics, this study provides a foundational framework for future research aimed at manipulating these interactions for agricultural benefits.

## 2. Materials and Methods

### 2.1. Plant Material

**Figure 1:**
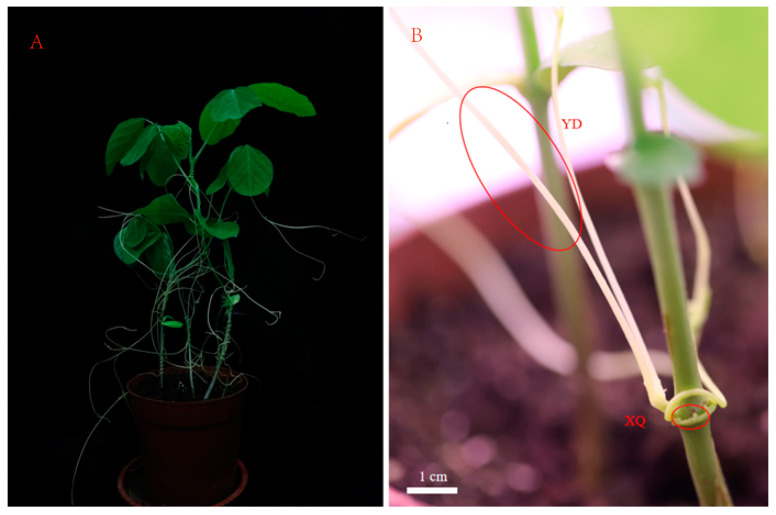
The phenotype and overall characteristics of *C. australis* parasitizing *G. max*. (**A**) An overview of *C. australis* parasitizing *G. max*. (**B**) A detailed view of *C. australis* parasitizing *G. max*. XQ is a close-up of the haustoria of *C. australis*, and YD is the distal stem segment of *C. australis*.

*Glycine max* (L.) *Merrill* seeds were provided by the Guizhou Provincial Institute of Agricultural Sciences, and *C. australis* was obtained from the College of Life Sciences, Ningxia University. The plants were planted in the greenhouse of the Physiological Regulation Laboratory of Guizhou Normal University (26°38′04″ N, 106°63′64″ E) from July to August 2023, with an average temperature of 26.2 °C. The greenhouse photoperiod was set to 16 h of light and 8 h of darkness. The beans were planted in the soil substrate of peat soil (the ratio of vermiculite to soil was 2:1), and the seedlings were watered with 1/2 of the nutrient solution every three days after they were unearthed. After 7 days of growth, the southern dodder seeds treated with sulfuric acid were planted at the base of the seedlings, and samples were taken after three weeks of culture. Sample collection: Take the sucker and stem of *C. australis* (see Figure 1, the distal end is the stem segment 4–8 cm away from the sucker), quickly put the collected sample into liquid nitrogen, and quench and then store it in a −80 °C freezer for later use.

### 2.2. LC-MS Analysis

Accurately weigh 60 mg of sample into a 1.5 mL centrifuge tube, and add two small steel balls and 600 μL of methanol-water (V:V = 7:3, including mixed internal standard, 4 μg/mL); Pre-cool in a −40 °C freezer for 2 min, then place in a grinder for grinding (60 Hz, 2 min); perform ultrasonic extraction in an ice water bath for 30 min, and static at −40 °C overnight; samples were centrifuged at 4 °C (12,000 rpm) for 10 min, 150 μL of supernatant was aspirated with a syringe, filtered using a 0.22 μm organic phase pinhole filter, and transferred to an LC injection vial and stored at −80 °C until LC-MS analysis. Quality control samples (QCs) were prepared by mixing the extracts from all the samples in equal volumes. All the extraction reagents in this experiment needed to be pre-cooled at −20 °C before use.

The mass spectrometry analysis was conducted using a liquid chromatography-mass spectrometry (LC-MS) system consisting of the Waters ACQUITY UPLC I-Class plus (Waters, Milford, MA, USA) coupled with the Thermo QE plus high-resolution mass spectrometer (Thermo Fisher Scientific, Waltham, MA, USA). The mass spectrometry parameters were as follows: capillary temperature at 320 °C; ion spray voltage at 3800 V (positive ion mode)/−3000 V (negative ion mode); scan range of 100–1200 *m*/*z*; full MS resolution of 70,000; auxiliary gas heater temperature at 350 °C; sheath gas flow rate at 35 (arbitrary units); S-lens RF level set to 50; MS/MS resolution of 17,500; and normalized collision energy (NCE)/stepped NCE set to 10, 20, and 40. An ACQUITY UPLC HSS T3 chromatographic column (100 mm × 2.1 mm, 1.8 µm) was used, with a column temperature of 45 °C. The mobile phase and gradient elution were established according to the description by Dai et al. [21]. The identification of compounds was based on multiple dimensions, including retention time (RT), accurate mass, secondary fragment ions, and isotopic distribution. The analysis was performed using the Human Metabolome Database (HMDB), LipidMaps (version 2.3), METLIN database, and the LuMet-Plant3.0 database.

The raw data were analyzed by using the metabolomics processing software, Progenesis QI v3.0 software (Nonlinear Dynamics, Newcastle, UK), and a combination of multi-dimensional analysis and single-dimensional analysis was used to screen the differential metabolites between the groups. In the OPLS-DA and PLS-DA analysis, the screening criteria were VIP values greater than 1 and a *p*-value value less than 0.05.

### 2.3. Total RNA Extraction and Transcriptomic Analysis

The total RNA from 6 samples was extracted using TRIzol^®®^ Reagent, and transcriptome libraries were constructed using the VAHTS Universal V5 RNA-seq Library Prep Kit. 150 bp paired-end sequencing was performed using the Illumina Novaseq 6000 platform, and 2,948,900 raw reads were obtained. DESeq2 was used for differentially expressed gene analysis, and R(v 3.2.0) was used for hierarchical cluster analysis to show the expression patterns. KEGG pathway analysis was carried out based on the hypergeometric distribution algorithm, the significance enrichment function was screened, and the enrichment analysis circle map was drawn with R (v 3.2.0). The sequencing data were annotated using the Glycine max genome (https://api.ncbi.nlm.nih.gov/datasets/v2/genome/accession/GCF_000004515.6/download?include_annotation_type=GENOME_FASTA&include_annotation_type=GENOME_GFF&include_annotation_type=RNA_FASTA&include_annotation_type=CDS_FASTA&include_annotation_type=PROT_FASTA&include_annotation_type=SEQUENCE_REPORT&hydrated=FULLY_HYDRATED, accessed on 24 February 2025). In this study, metabolomic analysis was conducted using 6 replicates, while transcriptomic analysis employed 3 replicates.

## 3. Results

### 3.1. LC-MS Metabolome Analysis of C. australis Aspirator and Distal Diameter Segments

**Figure 2:**
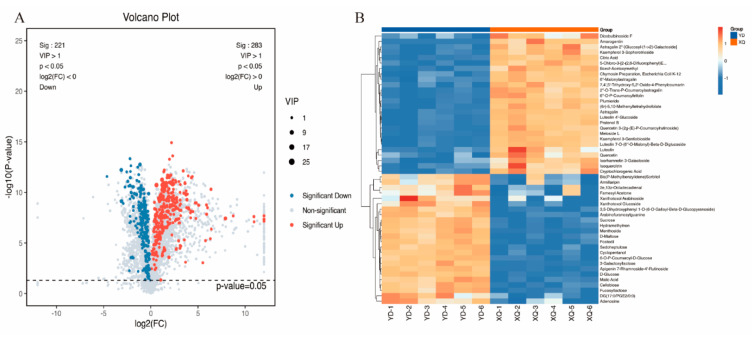
Analysis of differential metabolites in cumulators and stem segments of *C. australis*. (**A**) Volcano map VIP > 1 & *p*-value < 0.05 (**B**) cluster heatmap.

The absorption, accumulation, and transfer of metabolites from the sucker to the stem segment were studied, and the metabolites in the samples were detected by using LC-MS full scanning technology. A total of 5626 metabolites were detected during the growth and development of the sucker and stem of *C. australis*. PCA analysis showed that there were significant differences in the metabolite levels between the southern dodder aspirator and the distal diameter segment (Figure S1). Visualizing the VIP > 1 and *p*-value < 0.05 value via a volcano plot, 504 significantly differential metabolites were detected (Table S1), of which 283 were upregulated and 221 downregulated (Figure 2A). Hierarchical clustering of all significantly different metabolites revealed that the metabolites significantly upregulated in the aspirator included Diosbulbinoside F, Amarogentin, Astragalin 2”-[Glucosyl-(1->2)-Galactoside], Kaempferol 3-Sophorotrioside, and citric acid (Figure 2B).

### 3.2. Analysis of Differentially Expressed Genes in C. australis and Diameter

**Figure 3:**
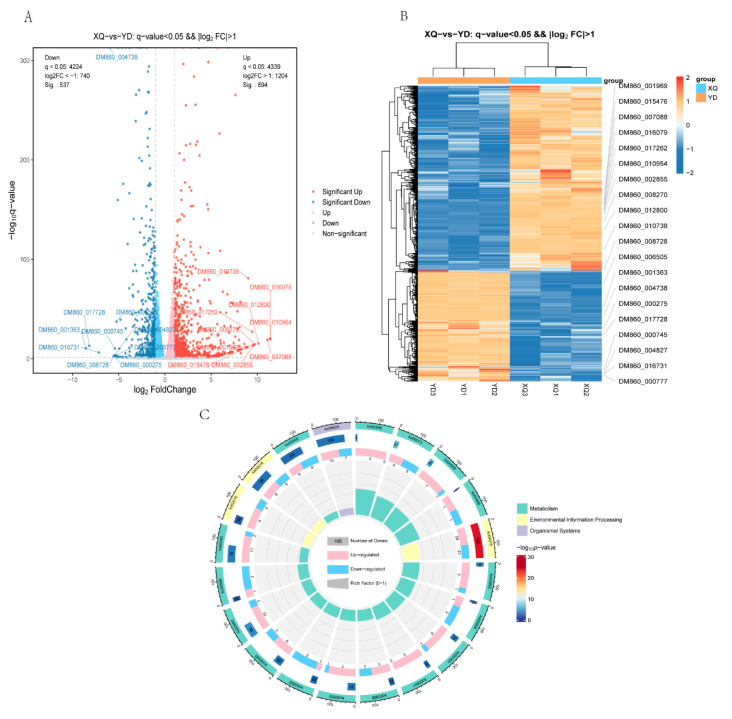
Screening and analysis of XQ and YD differential genes in *C. australis*. (**A**) Differentially expressed volcano diagram; (**B**) clustering diagram of differential gene grouping; (**C**) circle plot of differentially expressed genes and all genes in KEGG enrichment analysis.

To understand the relationship between the two tissue samples of the siphon and the distal diameter segment, and the difference in the gene expression between the different samples, we completed the parametric transcriptome sequencing of six samples (Tables S2 and S3), and via a volcano gram (Figure 3A). Differential expression gene analysis was performed using the DESeq2 software (1.46.0) where the genes meeting the thresholds of q-Value < 0.05 and fold change > 2 were defined as differentially expressed genes (DEGs). We found that 1204 genes were upregulated (894 of which were significantly upregulated) and 740 genes were downregulated (of which 537 were significantly downregulated) in the piper. The heat map (Figure 3B) shows that there were significant clustered differences in the gene expression between the samples of the XQ and YD groups, indicating that there were significant gene expression differences between the two groups. KEGG pathway enrichment analysis (Figure 3C) showed that there were significantly enriched KEGG pathway entries in the XQ and YD groups.

### 3.3. Differences in Metabolite and Gene Co-Expression Networks Between the Pipette and the Diameter End

**Figure 4:**
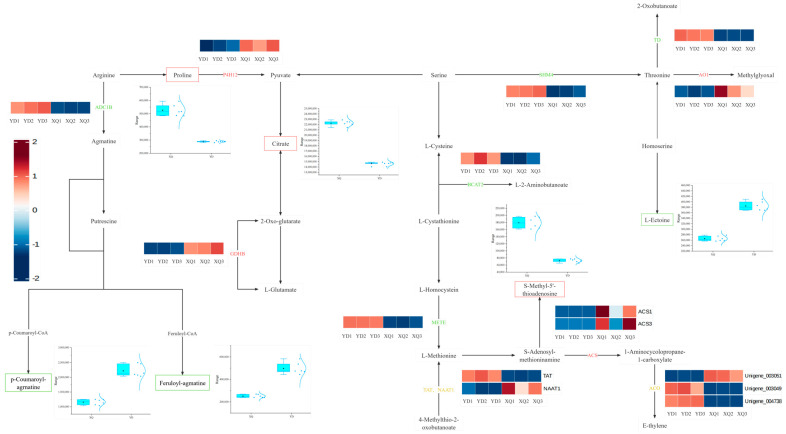
Changes in amino acid metabolism pathways and metabolites of related functional genes in the sucker and distal diameter segments. The red box represents higher levels of metabolites in the pipette, while the green box represents higher levels of metabolites in the distal diameter segment. Red represents upregulated genes or enzymes, green represents downregulated genes or enzymes, and yellow represents genes or enzymes that are both upregulated and downregulated. The cluster heatmap shows the cluster analysis of the related genes of the enzymes indicated in the pathway.

**Figure 5:**
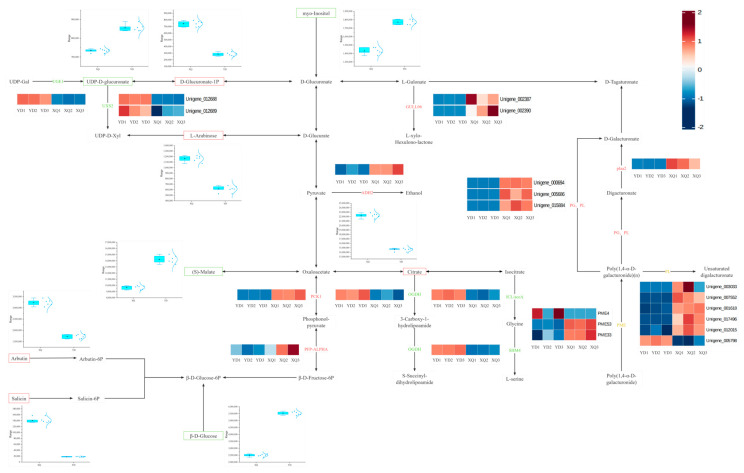
Changes in metabolites of carbohydrate metabolism pathways and related functional genes in the sucker and distal diameter segments.

Transcriptome and metabolome analysis showed that the contents of citric acid, L-arabinose, proline, D-glucuronic acid-1-phosphate and S-methyl-5′-thioadenosine were higher in the sucker fraction, while the contents of p-coumaroyl-spermine, feruloyl-spermine, inositol, β-D-glucose, UDP-D-glucuronic acid, and L-muramic acid were higher in the distal fraction (Figure 4). The expression of UDP-D-glucuronic acid in the sucker was downregulated, which may be regulated by the UGE1 gene, and the high expression at the distal end may promote the accumulation of metabolites (Figure 5). We also conducted a correlation analysis between the differentially expressed genes and differential metabolites in the dodder (Figure S2).

### 3.4. C. australis Seeds Communicate Through Genes with the Host

**Figure 6:**
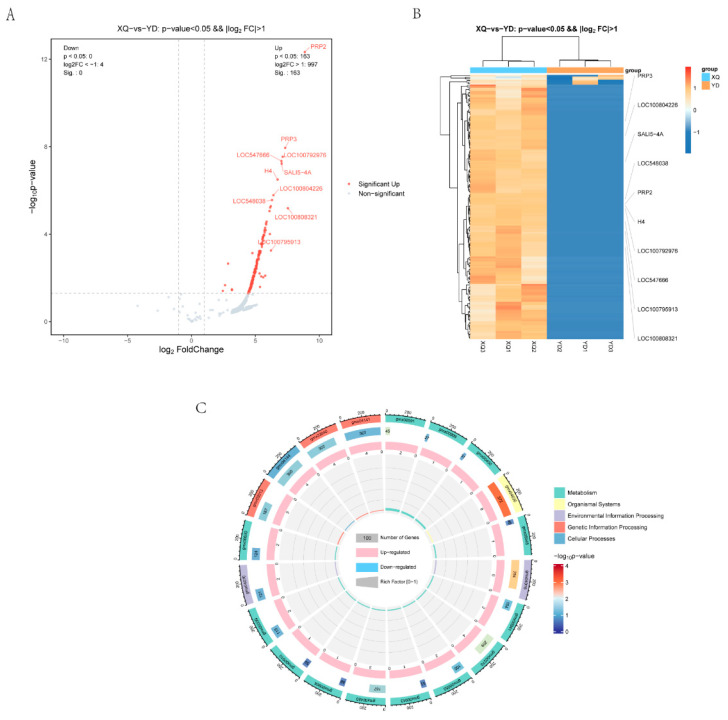
Differential gene screening and analysis of the absorption host (*G. max*) in *C. australis*. (**A**) Differentially expressed volcano diagram; (**B**) clustering diagram of differential gene grouping; (**C**) circle plot of differentially expressed genes and all genes in KEGG enrichment analysis.

By comparing the transcriptome sequencing results of *C. australis* with the genome of *G. max*, it was found that a total of 7110 genes were absorbed from the *G. max* sucker and stem, including 7001 genes in the sucker XQ and 109 genes in the stem (Table S4). The reads were aligned to the reference genome, and the alignment rate was 0.27~0.36% (Table S5). The analysis of the volcano map (Figure 6A) showed that 163 *G. max* genes were differentially expressed in the sucker, all of which were upregulated. The heat map (Figure 6B) showed that the gene expression levels in the XQ group were generally higher than those in the YD group, indicating that the gene expression in the sucker was active and related to the biological function of direct contact with the host. KEGG pathway enrichment analysis (Figure 3C) showed that there were significantly enriched KEGG pathway entries in the XQ and YD groups. We also conducted a correlation analysis between differentially expressed genes and differential metabolites in a dodder parasitizing *G. max* (Figure S3).

### 3.5. Differences in the Co-Expression Network of Genes and Metabolites in G. max Sucked by the C. australis Dodder Between the Sucker and the Distal Diameter Segment

**Figure 7:**
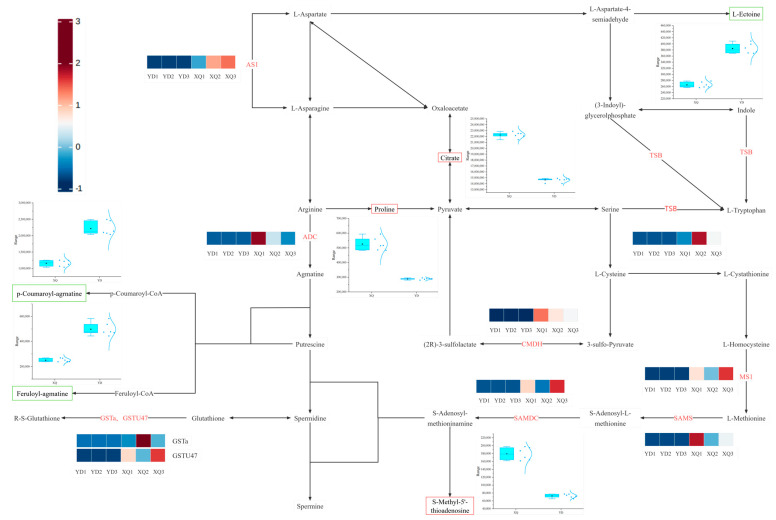
*C. australis* absorbs metabolites of *G. max* genes that regulate amino acid metabolism pathways in the sucker and distal stem segments. *C. australis* absorbs the relevant functional genes and metabolites in the amino acid metabolism pathway regulated by *G. max* gene regulation suckers and distal stem segments.

**Figure 8:**
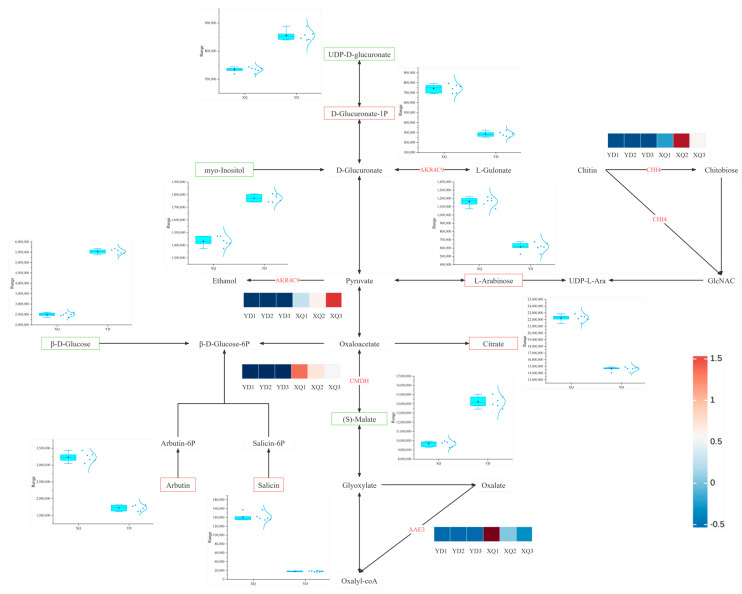
*C. australis* absorbs the *G. max* gene to regulate the suckers and related functional genes and metabolites in the distal stem segment that regulate the carbohydrate metabolism pathway.

After an in-depth comparative analysis of the genomes of *C. australis* and *G. max*, we found that 163 genes may be transferred from *G. max* to the sucker part of *C. australis* through horizontal gene transfer (HGT), a process that may lead to significant upregulation of the expression levels of these genes in the sucker. To further elucidate the relationship between the differential metabolites and differentially expressed genes between the inhaler and the distal diameter segment, we constructed a co-expression network of carbohydrates, amino acid metabolites, and the related genes, as shown in Figure 7 and Figure 8. Most of the differentially expressed genes in the sucker and distal diameter segments were upregulated in the sucker. The expression of differentially differentiated genes such as arginine decarboxylase (ADC), aspartate synthetase 1 (AS1), malate dehydrogenase (CMDH), phytoaldehyde ketone reductase (AKR4C9), and S-adenosylmethionine decarboxylase (SAMDC) in the sucker was significantly higher than that in the distal diameter segment. In the pipette part of dodder seeds, citric acid (Citrate), L-arabinose (L-arabinose), proline (Proline), D-glucuronate-1-phosphate (D-Glucuronate-1P), and S-Methyl-5′-thioadenosine were significantly higher than those in the distal fraction. In particular, the upregulation of S-methyl-5′-thiopadenosine expression may be regulated by SAMDC, S-adenosylmethionine synthetase (SAMS), and MS1 genes, and the high expression of these genes in the sucker may promote the accumulation of the corresponding metabolites. In contrast, in the distal part of dodder seeds, p-coumaroyl-agmatine, feruloyl-agmatine, (S)-Malate (S-malic acid), inositol (Myo-Inositol), the contents of β-D-glucose, β-D-glucose, uridine diphosphate-D-glucuronate, and L-muramic acid were higher than those in the pipeline. The upregulation of the expression of (S)-Malate may be regulated by the malate dehydrogenase (CMDH) gene, and the high expression in the sucker leads to the higher (S)-Malate in the distal diameter segment than in the aspirator, which is reflected by the box plot, which shows the comparison of their content in the aspirator and the distal diameter segment.

**Figure 9:**
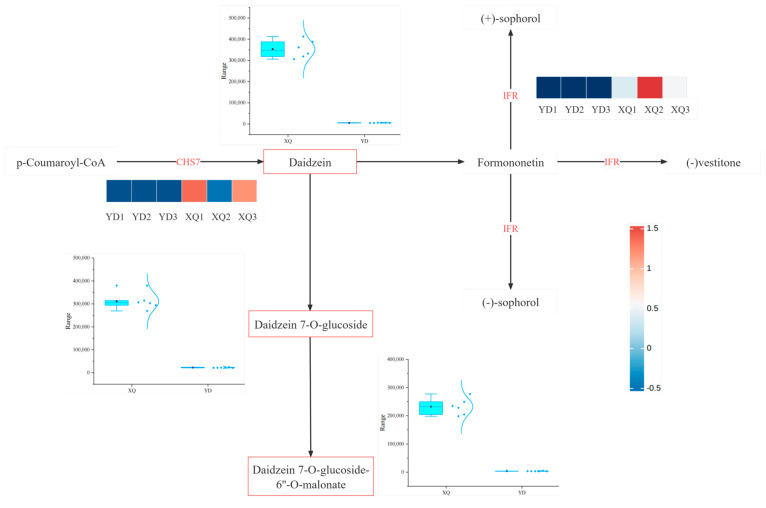
Co-expression network of isoflavone metabolites and related genes in the sucker and distal diameter segment after gene absorption from *G. max*.

By constructing a co-expression network of isoflavone metabolites and the related genes, as shown in Figure 9, we found that most of the differentially expressed genes in the sucker and distal diameter segments were upregulated in the sucker. For example, the expression of CHS and IRF was upregulated. Through the heat map, we observed the clustering of these differentially differentiated genes in the sucker and distal diameter segments, which further emphasized the significant expression differences in the genes involved in the metabolic pathway. The contents of Daidzein, Daidzein 7-O-glucoside, and Daidzein 7-O-glucoside-б”-O-malonate were significantly higher in the pipette part of *C. australis* than in the distal part. In contrast, the upregulation of CHS expression in *G. max* may lead to the production of Daidzein, Daidzein 7-O-glucoside, Daidzein 7-O-glucoside-6”-O-malonate, or direct aspiration by the suction part. Metabolites caused elevations of all three substances.

## 4. Discussion

*Cuscuta* is a holoparasitic plant that has no roots and leaves and is unable to photosynthesize. It is a valuable model plant for exploring plant–plant interactions and molecular transport [22]. It invades the host plant through the sucker to absorb the nutrients needed for growth and development. The formation of haustoria is induced by tactile, light, and chemical signals, involving complex molecular mechanisms, and there is also extensive bidirectional transport of proteins and RNA [23,24]. Studies have shown that cross-species transmission is essential for the adaptation, interaction, and evolution of parasitic plants [2,3,7,25]. With the advancement of omics techniques [21,26], it has become possible to analyze the transcriptomic information taken up by *C. australis* from the host *G. max* in the *G. max*–dodder subsystem by combining the genomic data from *C. australis* and *G. max*. The current studies mainly study the changes in metabolites in different organs of *C. australis* through metabolomics, the specificity of the sucker through transcriptomics, and the analysis of the relevant mechanisms of parasitic plants by combining metabolomics and transcriptomics [16,17,18]. Although there have been studies, the analysis of metabolite changes in the suckers and distal stems of *C. australis* has not been reported, and the molecular mechanism of specialization is still lacking.

### 4.1. Differential Analysis of Gene and Metabolite Expression in the Aspirator and Distal Stem of C. australis

The genes encoded by *C. australis* itself led to the metabolic differences between the sucker and the distal diameter segment, and the contents of L-arabinose (L-arabinose), Citrate, D-Glucuronate-1P (D-glucuronic acid-1-phosphate), and proline were higher in the sucker. L-arabinose is one of the main components of plant cell walls, and the high content of L-arabinose in *C. australis* seed aspirators may be related to the synthesis and maintenance of its cell wall, especially in the region of the aspirator connected to the host [27]. D-Glucuronate-1P may be involved in energy metabolism and substance transport. The high content of D-Glucuronate-1P in the pipette may reflect the high demand for energy and material transport. The downregulation of UGE1 may lead to the downregulation of UDP-D-glucuronate, which leads to the upregulation of D-Glucuronate-1P. In rice, the loss of function of BP1 (encoding UDP-glucose-4-epimerase) leads to a large accumulation of UDP-glucose and an imbalance of other UDP sugars, which further confirms the role of UGE1 in UDP-D-glucuronate metabolism and its importance in plant development [28].

The increase in proline content in the haustoria of the dodder may be due to the exchange of substances between the haustoria and soybean. There is an extensive exchange of materials and signals between the dodder and its host plants, including water, nutrients, nucleic acids (such as lncRNA), and proteins. This exchange of substances may cause a portion of the proline synthesized by soybean to be transferred to the haustoria of the dodder [29]. Liu et al. used proteomics to analyze the proteins transferred between host plants and a dodder. In the soybean–dodder system, they identified more than 1674 dodder proteins in soybean stems and 1547 soybean proteins in dodder stems, indicating that the dodder exchanges substances with soybean through haustoria. This may affect the distribution of metabolites in the aspirator and distal stems [30]. The elevated citric acid content may be due to the upregulation of PCK1 (phosphoenolpyruvate carboxykinase 1). The acetylation of PCK1 inhibits its gluconeogenesis reaction and facilitates the synthesis of oxaloacetate (OAA) from phosphoenolpyruvate, which benefits the tricarboxylic acid (TCA) cycle and thereby affects the production of metabolites such as Citrate [31].

### 4.2. Differential Genes Taken by C. australis Chinensis from G. max Hosts

*C. australis* seeds receive nutrients and solutes from *G. max*, and the expression of genes related to the transport of many nutrients increases during the sucker stage of dodder seeds [32]. It has been found that *C. australis* europaea parasitism may affect the amino acid transport and release of broad bean stems [33]. In this study, it was found that when *C. australis* was parasitic on *G. max* and absorbed some genes from *G. max*, it also affected the amino acid metabolism of *C. australis* to a certain extent, resulting in differences between the distal and proximal ends of *C. australis* seeds. Citrate, proline, and S-Methyl-5′-thioadenosine were more abundant in the pipette part. The upregulation of organic acids may be related to energy metabolism and material transport, especially the citric acid cycle, which is a key intracellular energy metabolism pathway, and the upregulation of its related metabolite, Citrate, reflects the high energy demand of the sucker [34]. The content of the proline in the sucker part of the dodder is also higher than that at the distal end. Proline maintains an osmotic balance of the cytosol to alleviate cellular damage while also scavenging reactive oxygen species (ROS) to protect nucleic acids and proteins and is a rapid source of compensation for nitrogen, carbon, and reducing capacity [35]. It has also been shown that *C. australis* can also regulate the nitrogen (N) uptake activity [36]. We hypothesize that the proline content of the inspirator fraction is higher than that of the distal fraction, possibly since the inspirator fraction assumes the task of regulating host nitrogen activity. S-methyl-5′-thioadenosine (MTA) is a nucleoside (Methylthioadenosine) produced by S-adenosylmethionine (SAM) during polyamine biosynthesis, and the activity and expression level of SAMDC directly affect the efficiency of the conversion of S-adenosylmethionine SAM to decarboxylated dSAM [37], which in turn affects the production of MTA. Therefore, we suspect that the significant expression of SAMDC in the aspirator fraction will result in more SAMs being converted to dSAM, resulting in more MTA in the aspirator fraction.

Chalcones synthase (CHS) is one of the key enzymes in the secondary metabolic pathway of plants, which catalyzes the synthesis of chalcones, which are the first steps in the synthesis of flavonoid compounds, including isoflavones. CHS forms chalcones by polymerizing three molecules of malonyl-CoA (malonyl-CoA) and one molecule of shikimic acid (p-Coumaroyl-CoA). Daidzein is a major isoflavone found mainly in *G. max* [38]. In an article investigating the biosynthesis of 5-deoxy isoflavones (e.g., genistein and its conjugates) in *G. max* [39], it was found that CHS plays a key role in the initial step in the biosynthesis of daidzein, i.e., the generation of chalcone. Chalcone is then catalyzed by CHR and other enzymes to produce daidzein. As can be seen in the figure, the content of the metabolite, daidzein, in the sucker part is higher, and since the content of CHS in the sucker part of *C. australis* is higher than that of the distal end, we surmise that it may be due to the increase in the content of daidzein in the gene CHS absorbed by the dodder from the host *G. max*, which leads to the increase in the daidzein content. However, it is also possible that the metabolite content is elevated due to the direct absorption of some metabolites by the pipette. Isoflavone reductase (IFR) is involved in the final step of the biosynthesis of various isoflavones in daidzein [40].

## 5. Conclusions

In this study, metabolomics and transcriptomic analysis were used to compare the differences between the sucker part and the distal part of *Cuscuta australis* R. Br. It is parasitic on *G. max* and absorbs some genes from *G. max*, revealing the differences in metabolism and gene expression between the sucker and stem of *C. australis* during the parasitism. It was found that a total of 163 genes in *G. max* were absorbed by the sucker, and the expression of genes involved in glycolysis, secondary metabolism, and hormone signal transduction in the sucker was significantly upregulated, while the expression in the stem was downregulated. Metabolomic analysis showed that the contents of citric acid, proline, and S-methyl-5′-thioadenosine were higher in the dodder sucker, suggesting that the increased energy and nitrogen requirements of the suckers may be related to their high energy requirements and the function of regulating host nitrogen activity. KEGG enrichment analysis showed that the significant enrichment of the isoflavone metabolism pathway may be due to the absorption of CHS and IRF from the host *G. max* by the sucker part, resulting in a significant difference in the isoflavone metabolic distribution between the distal end of the *C. australis* dodder and the sucker. This study provides an important reference for the study of the growth and development, parasitic mechanism, and the development of prevention and control strategies of *C. australis*.

## Data Availability

All sequencing data are available through the NCBI Sequence Read Archive under the accession number PRJNA1202882; other data are available from the manuscript and supplementary files.

## Supplementary Materials

The following supporting information can be downloaded at: https://www.mdpi.com/article/10.3390/metabo15030172/s1, Figure S1: PAC plot; Figure S2: Correlation Analysis between Differentially Expressed Genes and Differential Metabolites in Dodder; Figure S3: Correlation Analysis between Differentially Expressed Genes and Differential Metabolites in Dodder Parasitizing *G. max*; Table S1: Differential metabolites; Table S2: Sequencing data quality preprocessing results; Table S3: Statistical results of alignment rate with reference genome; Table S4: Statistical results of alignment rate with reference genome; Table S5: Statistical results of transcriptome sequencing of *Cuscuta* seeds and *G. max* reference genome alignment rate.
