# Supplementary material for: Molecular Mechanism of Cuscuta Haustorium Specialization Inferences from Transcriptome and Metabolome Analysis
Source: Metabolites. 2025 Mar 3;15(3):172. doi: 10.3390/metabo15030172 (PMC11943614; doi:10.3390/metabo15030172)
Supplement: Supplementary file 1 [file metabolites-15-00172-s001.zip › Supplementary figures and tables.pdf]

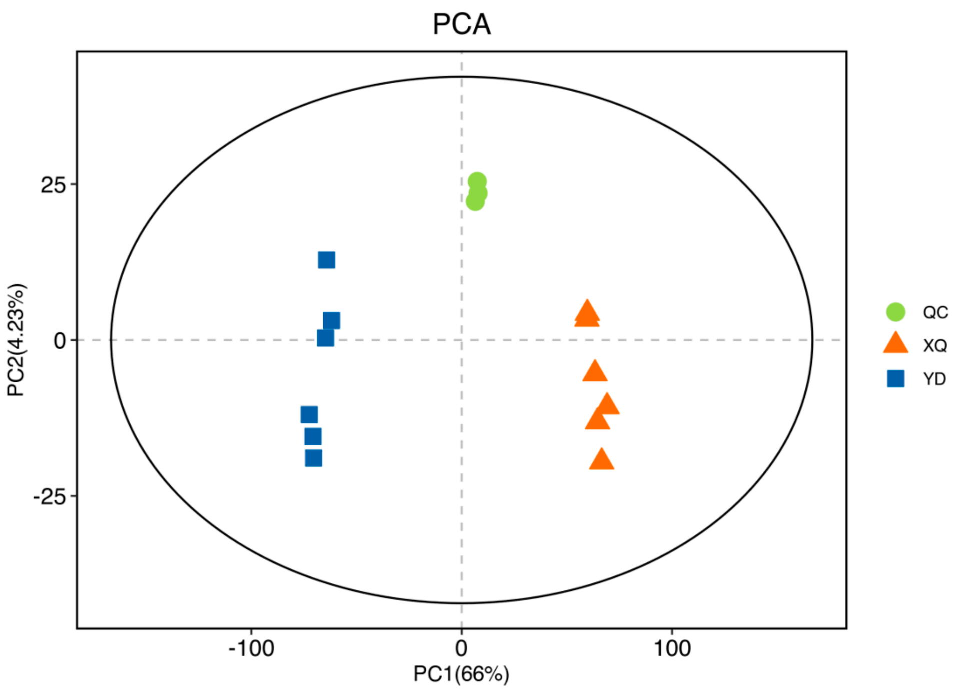

Supplementary Figure S1. PCA plot.

Supplementary Table S2. Sequencing data quality preprocessing results.

Sample	Raw Reads(M)	Raw Bases(G)	Clean Reads(M)	Clean Bases(G)	ValidBases(%)	Q30(%)	GC(%)
XQ1	48.34	7.04	46.47	6.77	96.13	94.3	47.08
XQ2	50.08	7.3	48.23	7.03	96.31	95.04	47.04
XQ3	49.27	7.16	47.5	6.9	96.4	95.56	48.54
YD1	49.95	7.09	46.81	6.65	93.72	94.5	46.85
YD2	50.9	7.37	48.59	7.03	95.46	94.49	46.83
YD3	46.35	6.78	44.77	6.55	96.6	94.39	46.98

Supplementary Table S3. Statistical results of alignment rate with reference genome.

Sample	XQ1	XQ2	XQ3	YD1	YD2	YD3
**Total reads**	46467964	48227394	47499360	46813128	48587288	44774182
**Total mapped reads**	45402927 (97.71%)	47111107(97.69%)	46385267 (97.65%)	45755931 (97.74%)	47478483 (97.72%)	43755165 (97.72%)
**Multiple mapped**	1340903(2.89%)	1403374(2.91%)	1431990(3.01%)	1355970 (2.90%)	1411979 (2.91%)	1299881(2.90%)
**Uniquely mapped**	44062024 (94.82%)	45707733 (94.78%)	44953277 (94.64%)	44399961 (94.85%)	46066504 (94.81%)	42455284 (94.82%)
**Read-1**	22049756 (47.45%)	22852455 (47.38%)	22460658 (47.29%)	22218438 (47.46%)	23053967 (47.45%)	21254245 (47.47%)
**Read-2**	22012268 (47.37%)	22855278 (47.39%)	22492619 (47.35%)	22181523 (47.38%)	23012537 (47.36%)	21201039 (47.35%)
**Reads map to '+'**	22017276 (47.38%)	22840153 (47.36%)	22466041 (47.30%)	22189649 (47.40%)	23021830 (47.38%)	21216832 (47.39%)
**Reads map to '-'**	22044748 (47.44%)	22867580 (47.42%)	22487236 (47.34%)	22210312 (47.44%)	23044674 (47.43%)	21238452 (47.43%)
**Non-splice reads**	28340011(60.99%)	29450227 (61.07%)	28425954 (59.84%)	28985538 (61.92%)	29739280 (61.21%)	27284976 (60.94%)
**Splice reads**	15722013 (33.83%)	16257506 (33.71%)	16527323 (34.79%)	15414423 (32.93%)	16327224 (33.60%)	15170308 (33.88%)
**Reads mapped in proper pairs**	43765440 (94.18%)	45432006 (94.20%)	44753514 (94.22%)	44121054 (94.25%)	45762188 (94.19%)	42157020 (94.15%)

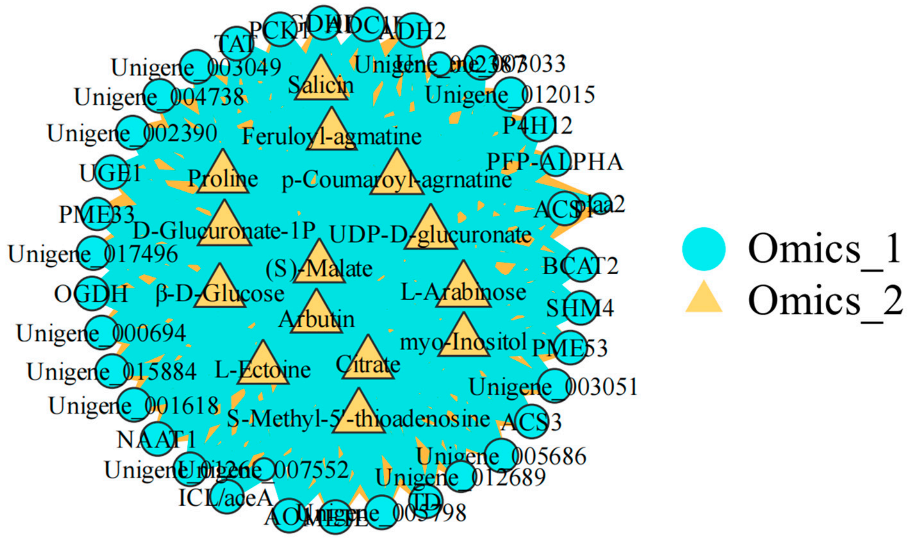

Supplementary Figure S2. Correlation Analysis between Differentially Expressed Genes and Differential Metabolites in Dodder.

Supplementary Table S4. The number of genes detected is partially counted.

Sample	Gene_num
XQ1	3039
XQ2	3236
XQ3	3156
YD1	38
YD2	35
YD3	36
Total	7110

Supplementary Table S5. Statistical results of transcriptome sequencing of Cuscuta seed and soybean reference genome alignment rate.

Sample	XQ1	XQ2	XQ3	YD1	YD2	YD3
**Total reads**	46467964	48227394	47499360	46813128	48587288	44774182
**Total mapped reads**	143829(0.31%)	158069(0.33%)	171407(0.36%)	135102(0.29%)	137964(0.28%)	122251(0.27%)
**Multiple mapped**	68739(0.15%)	75557(0.16%)	82024(0.17%)	70131(0.15%)	69418(0.14%)	59821(0.13%)
**Uniquely mapped**	75090(0.16%)	82512(0.17%)	89383(0.19%)	64971(0.14%)	68546(0.14%)	62430(0.14%)
**Read-1**	38684(0.08%)	42353(0.09%)	45742(0.10%)	32726(0.07%)	34871(0.07%)	31909(0.07%)
**Read-2**	36406(0.08%)	40159(0.08%)	43641(0.09%)	32245(0.07%)	33675(0.07%)	30521(0.07%)
**Reads map to '+'**	37050(0.08%)	40774(0.08%)	44437(0.09%)	32451(0.07%)	34283(0.07%)	31450(0.07%)
**Reads map to '-'**	38040(0.08%)	41738(0.09%)	44946(0.09%)	32520(0.07%)	34263(0.07%)	30980(0.07%)
**Non-splice reads**	36134(0.08%)	38531(0.08%)	41356(0.09%)	28458(0.06%)	29991(0.06%)	28468(0.06%)
**Splice reads**	38956(0.08%)	43981(0.09%)	48027(0.10%)	36513(0.08%)	38555(0.08%)	33962(0.08%)
**Reads mapped in proper pairs**	62868(0.14%)	69152(0.14%)	76008(0.16%)	55908(0.12%)	55092(0.11%)	48824(0.11%)

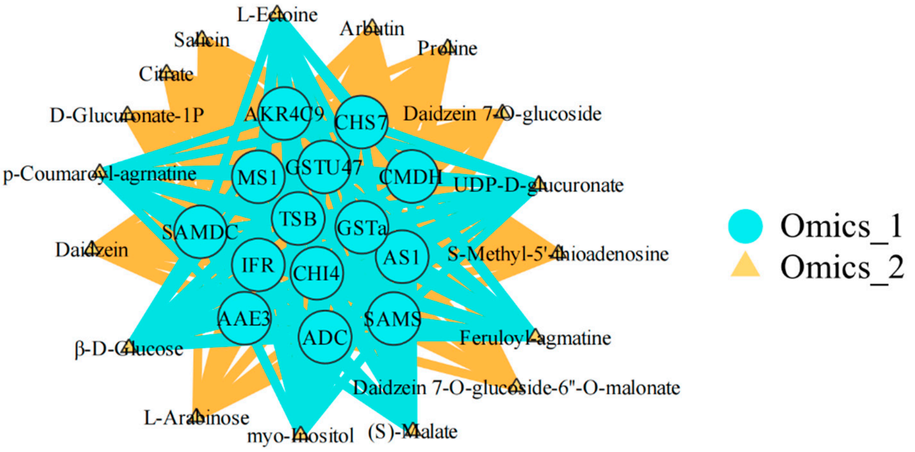

Supplementary Figure S3. Correlation Analysis between Differentially Expressed Genes and Differential Metabolites in Dodder Parasitizing ***G.max***.
